# Enhancing awareness of vitiligoid vulvar lichen sclerosus: An underrecognized variant in patients with skin of color

**DOI:** 10.1016/j.jdcr.2024.08.017

**Published:** 2024-08-31

**Authors:** Shivani Desai, Rachel Blasiak, Jayson Miedema, Sarah B. Corley

**Affiliations:** a University of North Carolina School of Medicine, Chapel Hill, North Carolina; b Department of Dermatology, University of North Carolina School of Medicine, Chapel Hill, North Carolina

**Keywords:** lichen sclerosus, vitiligo, vulva

## Introduction

Characterized by skin changes of depigmentation, vitiligoid vulvar lichen sclerosus (VVLS) often goes undiagnosed or misdiagnosed as vitiligo in patients with skin of color. A clinical suspicion of vulvar vitiligo (VV) may not be worked up histopathologically, missing the diagnosis of vulvar lichen sclerosus (VLS) and thereby leading to architectural changes and risk of vulvar squamous cell carcinoma. Thus, recognition of this variant in patients with skin of color is imperative to provide adequate long-term treatment to decrease these risks.

It can be difficult to distinguish VVLS from VV clinically as both conditions present with depigmentation. VLS may present with pruritus, burning, dyspareunia, and texture changes, whereas VV is asymptomatic.^1^ VLS is also differentiated by architectural changes present in some patients, which are not present in patients with VV. Additionally, it is important to acknowledge the coexistence of VLS and VV to prevent missing a diagnosis of concomitant VLS in a patient with vitiligo.^1^ Therefore, current recommendations in the literature support assessing for findings such as fissures, lichenification, atrophy, erosions, and petechiae in patients with VV as these findings may indicate concomitant VLS.^1^

Understanding the distinction between VVLS and VV is vital because treatment of VLS is necessary to mitigate long-term sequelae. Although both conditions may be treated with high-potency topical steroids or calcineurin inhibitors, there are distinctions in treatment timelines.^2^^,^^3^ VV is typically treated episodically whereas VLS is treated with an initial treatment plan followed by a life-long maintenance treatment as preventive use of topical steroids has shown to decrease the risk of developing vulvar squamous cell carcinoma and decrease further scarring of the vulva.^3^

Our goal is to increase recognition of this variant of VLS in adult patients with skin of color and its similarity to VV. Here, we present 3 cases of biopsy-supported VVLS.

## Case presentations

### Case 1

**Fig 1:**
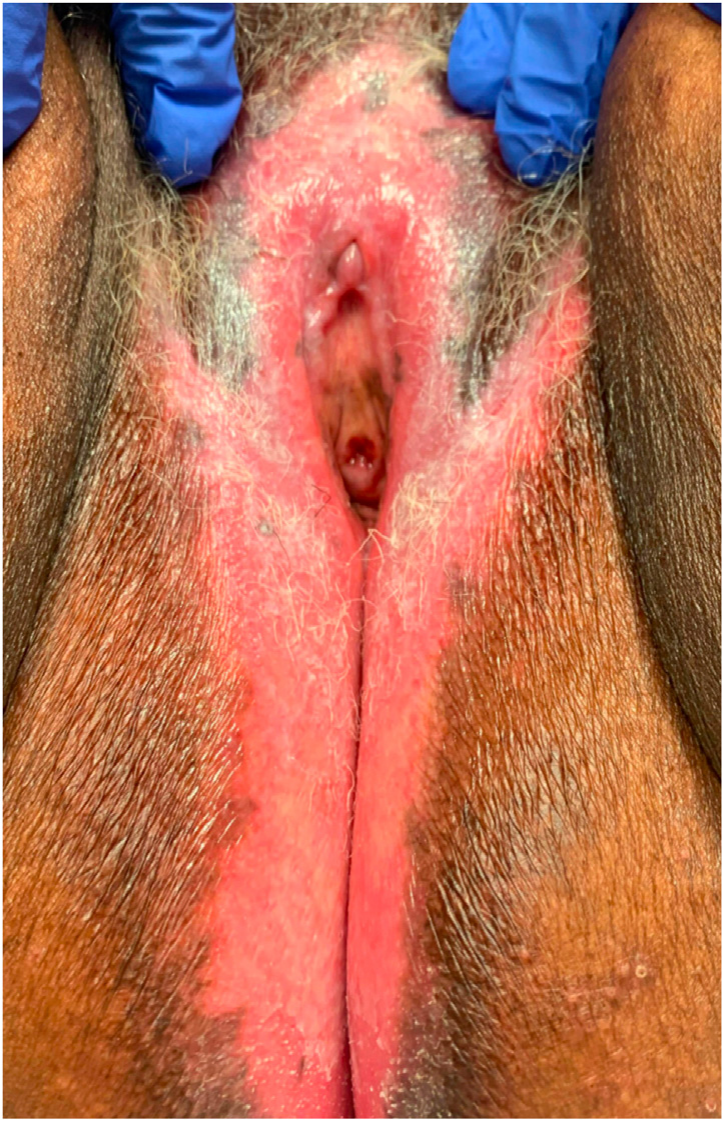
Case 1. Vulvar lichen sclerosus. Depigmented patches of the vulva in case 1.

A 73-year-old Black woman presented to an outside dermatology clinic with depigmentation and pruritus of the vulva. She had initially been diagnosed with vitiligo and treated with tacrolimus 0.1% ointment and triamcinolone 0.1% ointment. Without significant improvement over time, a 4-mm punch biopsy was performed showing papillary dermal sclerosis, perivascular and interstitial lymphocytic infiltrate involving the reticular dermis leading to a diagnosis of lichen sclerosus. The diagnosis was revised from vitiligo to VVLS. She was initiated on clobetasol 0.05% ointment with improvement, but not resolution, of her pruritus. Her care was transitioned to our vulvar dermatology clinic, and she was noted to have depigmented patches and erythema involving the labia majora, clitoral hood, perineum, and anus with fissures at the clitoral hood in addition to some mild hyperkeratosis at the buttocks (Fig 1). Additionally, she was found to have partial agglutination of the labia and partial clitoral phimosis. She was initiated on halobetasol 0.05% ointment with resolution of the pruritus and improvement of pigmentary changes.

### Case 2

**Fig 2:**
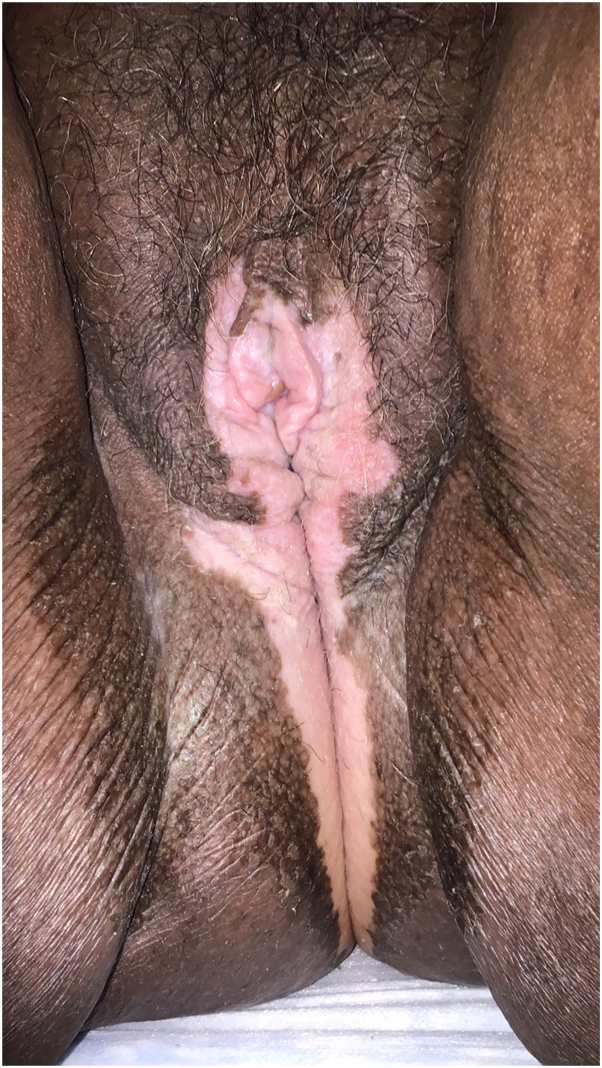
Case 2. Vulvar lichen sclerosus. Depigmented patches of the vulva in case 2.

A 72-year-old Black woman presented to her gynecologist with pruritus and depigmentation of the vulva. She was diagnosed with VV based on initial clinical impression. However, with persistent pruritus, a 4-mm punch biopsy of the left labium majus was performed revealing lichen sclerosus. The diagnosis was revised from VV to VVLS and she was referred to our vulvar dermatology clinic. On examination, she was noted to have diffuse depigmentation of the labia minora, labia majora, and perineum in addition to hyperkeratotic plaques on the right labium minus (Fig 2). She was found to have partial agglutination of the labia minora, inability to retract the clitoral hood, and narrowing of the introital aperture. She was initiated on halobetasol 0.05% ointment twice daily for 3 months with resolution of her intense pruritus, resolution of the hyperkeratotic plaques, and improvement of the depigmentation. She eventually transitioned to triamcinolone 0.1% ointment daily for maintenance.

### Case 3

**Fig 3:**
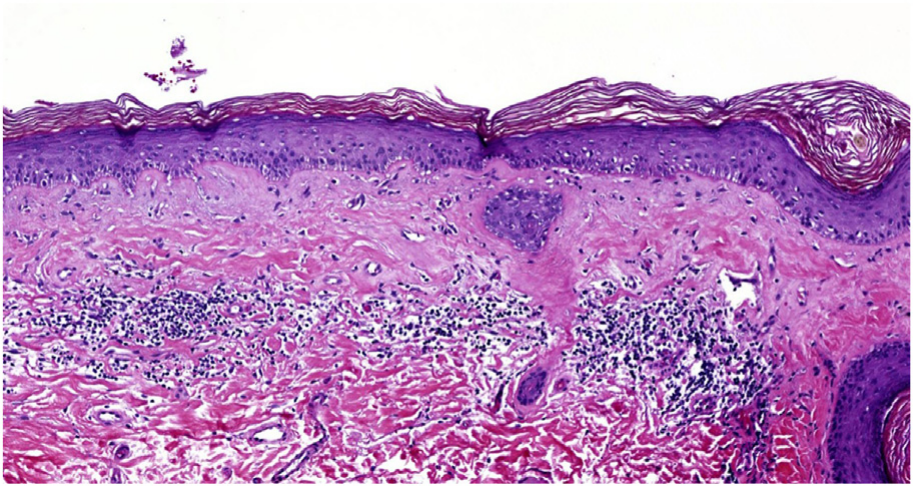
Vulvar lichen sclerosus, biopsy of dermis hyalinization with an edematous band of collagen overlying a lymphocytic inflammatory infiltrate in case 3. Hematoxylin and Eosin, medium magnification (100×)

A 58-year-old Black woman with a history of pernicious anemia presented to an outside dermatology clinic with a several week history of pruritus and depigmentation of the labia majora. She was prescribed topical ruxolitinib for a presumed clinical diagnosis of vitiligo. The patient endorsed the depigmentation spreading with persistence of the pruritus and thus was referred to our vulvar dermatology specialty clinic. On examination, she was noted to have depigmented patches along the clitoral hood, medial labia majora and labia minora extending to the perineum, and medial buttocks. No atrophy or architecture changes were noted. Because of concern of lichen sclerosus given the symptom of pruritus, a snip biopsy of the posterior aspect of the left labium majus was performed. Biopsy showed the dermis was hyalinized with an edematous band of collagen overlying a lymphocytic inflammatory infiltrate consistent with lichen sclerosus (Fig 3). A SOX10 stain was performed demonstrating a reduction of melanocytes. The diagnosis was revised from VV to VVLS and she was initiated on halobetasol 0.05% ointment nightly for 2 months with complete resolution of the pruritus and partial repigmentation on follow up and thus the medication frequency was decreased to 3 times a week for maintenance.

## Discussion

VLS is a chronic inflammatory dermatosis commonly presenting with pruritus and atrophic pale plaques that may result in sequalae of scarring, architectural changes, dyspareunia, and increased risk of vulvar squamous cell carcinoma. In skin of color, VLS may present with depigmentation rather than hypopigmentation or pallor thus mimicking vitiligo.^4^ VLS does not currently have a definitive pathogenesis but has supporting autoimmune etiology with a bimodal incidence in prepubescent girls and menopausal women.^5^ A diagnosis of VLS may be achieved based on clinical history however with diagnostic uncertainty or atypical features, histopathology should be performed for confirmation. Histologically confirmed VLS may include findings of epidermal hyperkeratosis, dermal sclerosis, and a band-like inflammatory lymphocytic infiltrate within the dermis.^6^ Because of the risk of scarring and squamous cell carcinoma associated with VLS, it is imperative for physicians to recognize this variant to ensure effective long-term management.^3^^,^^5^

In this case series, all 3 patients were presumed to have a diagnosis of VV and were started on treatment that resulted in refractory symptoms of pruritus of the vulva (Table I). All patients subsequently received a histopathologic confirmed and revised diagnosis of VVLS. Specifically, in the third case, a SOX10 stain was performed revealing a reduction of melanocytes supporting a clinicopathologic diagnosis of VVLS. VLS resembling vitiligo has previously been reported in the literature in patients with vitiligoid lesions with histologically proven lichen sclerosus.^7^ The coexistence of lichen sclerosus and vitiligo in some patients may be explained by both conditions being driven by autoimmune etiology.^7^ Further, it has been suspected that lichenoid inflammation may prompt an autoimmune response against melanocytes.^7^^,^^8^ In a review of 266 patients with lichen sclerosus and VV, 15 patients were found to have VVLS, 3 of which occurred in the genital area.^7^ Although not found to be the case in the patients presented above, the coexistence of VLS and VV may also be observed and should be considered should symptoms develop such as pruritus, sensitivity, and dyspareunia in a patient with a known diagnosis of vitiligo.

**Table I:** Clinical findings

Case	Symptom presentation	Initial clinical diagnosis	Biopsy-confirmed diagnosis	Treatment
1	Depigmentation and pruritus	Vulvar vitiligo	Vulvar lichen sclerosus	Halobetasol 0.05% ointment
2	Depigmentation and pruritus	Vulvar vitiligo	Vulvar lichen sclerosus	Halobetasol 0.05% ointment Triamcinolone 0.1% ointment
3	Depigmentation and pruritus	Vulvar vitiligo	Vulvar lichen sclerosus	Halobetasol 0.05% ointment

Although VVLS may present with architectural changes, it is important to note that in case 3, there were no architectural changes noted at the initial presentation. This case underscores the need to acknowledge symptoms in the absence of architectural changes and pursue biopsy with uncertainty regarding diagnosis. Our work highlights the need to raise awareness to avoid misdiagnosis in patients with skin of color because currently there is a gap in the literature regarding this variant of VLS in the adult population as it has primarily been reported in the pediatric population.^5^

Our cases demonstrate the need for physician recognition of vulvar symptoms and signs to delineate VLS versus VV when patients with skin of color present with depigmented patches on the vulva, perineum, and perianal area. Enhancing awareness of the variant, VVLS, can significantly contribute to improving the accuracy of the diagnosis. This is especially important because patients who are compliant with long-term topical corticosteroid therapy for VLS have significant differences in architecture changes and incidence of vulvar carcinoma.^3^ The cases we presented in this series demonstrate the similarity in appearance of VV and VVLS that may result in diagnostic and treatment delay.

## Conclusion

The cases presented in this report underscore the need for physician recognition of the similarities between VLS and VV and existence of the variant VVLS. Particular attention to these diagnoses should be given to patients with skin of color with presentations similar to our described cases.

## Conflicts of interest

None disclosed.
